# Problems With Police Reports as Data Sources: A Researchers' Perspective

**DOI:** 10.3389/fpsyg.2020.582428

**Published:** 2020-10-22

**Authors:** C. Dominik Güss, Ma. Teresa Tuason, Alicia Devine

**Affiliations:** ^1^Department of Psychology, University of North Florida, Jacksonville, FL, United States; ^2^Department of Public Health, Clinical Mental Health Counseling, University of North Florida, Jacksonville, FL, United States

**Keywords:** police reports, data, ethics, quality, validity, biases

## Introduction

Police officers are at the forefront of dealing with crimes. As one part of their work they are required to write reports, such as crime case reports, traffic collision reports, driving under the influence reports, or death case reports about those crimes. These reports document information regarding an investigation and are often later used as legal documents for law enforcement and outside agencies such as insurance companies. In extreme cases, a police report could even make its way to the Supreme Court. Thus, a police report should be detailed and contain accurate information about an incident or crime (e.g., “factual, accurate, clear, concise, complete, timely,” Sacramento State Police Department, 2014, p. 3).

One would expect then that police reports would offer a wealth of information that researchers might be able to use for conducting their projects. Yet, there are several problems related to using police reports as potential data source. The following comments are based on the experiences of the authors while they were researching gun violence in the United States (a follow up to a prior study, Tuason and Güss, 2019) after contacting over 300 police stations nationwide requesting police reports for specific cases. This was their first project working with police reports as a data source (large-scale multi-jurisdictional approach). Hopefully, our shared experiences will help other researchers when collecting data and utilizing police reports.

Specifically, although our experience was that police departments were professional and helpful, we identified several problems with police reports in the United States of America. Every country has different laws and procedures, so what we mention here might not apply in other countries. In other countries, some of the same challenges may be relevant, others may not apply, and some additional challenges not mentioned here may be encountered.

## Police Reports May not Be Released

As naïve researchers we expected police reports to be public records that are accessible under state laws such as Public Records Law (for example Illinois Compiled Statutes ILCS, 5 ILCS 140), Sunshine Law in Florida (Florida Statutes, Title XIX, Chapter 286 and Title X, Chapter 119), Right-to-Know Law in Pennsylvania (Commonwealth of Pennsylvania Right to Know Law, Act 3 of 2008), similar to the Freedom of Information Act (FOIA, 5 U.S.C. § 552) which allows the public to seek records from any federal organization. In many cases, however, after contacting police departments, we did not get the reports we asked for. Out of 320 police reports requested so far, we have received only 95 (with responses obtained after varied amounts of time due to different state laws). In some instances, a police department did not return communication requests at all.

One reason—and understandably so—is that a specific case is still pending in court. Therefore, the information cannot be simply shared.

- “Your request must be respectfully denied. The records that you requested are exempt from disclosure because they are related to a criminal investigation. Section 708 (b) (16) of the RTKL [Right-to-Know Law] exempts from disclosure a “record of an agency relating to or resulting in a criminal investigation, including…(ii) Investigative materials, notes, correspondence, videos and reports.”” (PA)

Another reason is that each state has its own laws about sharing police reports. Here are some examples of denials we received from police departments in different states:

- “…I must deny access to these records on the basis of Public Officers Law Section 87(2) (b) as such information, if disclosed, would constitute an unwarranted invasion of personal privacy.” (NY)
- “…The City is only required to provide public records to the citizens of the state of Alabama.” (AL)
- “Pursuant to Section 6254 of the Government Code, you must be named as a victim or be an authorized representative of the victim on the report.” (CA)

## Obtaining Police Reports is a Tedious Process

Another unpleasant surprise for a researcher is the convoluted process related to acquiring these police reports. The first step is to find the responsible police department; where the crime happened, and which police department has jurisdiction and is responsible for reporting. Depending on the police department, a researcher may need to contact several people or sub-departments even to find out who deals with releasing reports. Not everyone who works for the police department may know their policies on releasing reports. Sometimes email communication is not enough or there may not be an option for email communication. The researcher might have to fill out an online form, or might need to make a phone inquiry first, or might have to send in a request letter by regular mail. For some departments, the researcher might have to pay certain costs related to processing such a request by sending a money order first. Sometimes it is unclear what the costs are because they depend on the number of copies. Then, one has to liaise with the police department to learn about the costs, and make the payment, before the request can be made. In brief, the whole process is more complicated than one might think.

## Police Reports do not Provide a Complete Data Set

Many police reports are incomplete for at least four reasons. First, some police stations claim that they have two types of reports: one that are for internal use only and is not shared with the public, and another that they could share. Second, when the document is shared, they do not document all the variables in which a researcher might be interested. In the present study, the police reports received ranged from 3 to 182 pages (one file had 589 pages). Obviously, the short reports lack information that might be important for researchers, for example, shooter characteristics such as socio-economic status or marital status. For example, one police report stated, “I did not conduct any interviews with the victims, witnesses, or the suspect in this case.” Or another one that was brief and showed the lack of information, “After canvassing the scene, there were no cooperating witnesses that would provide any information;” What exactly the argument was about was not mentioned. A third reason for the inconsistencies in the report content is related to the fact that data collection practices are often not uniform across the multiple jurisdictions in the nation.

Fourth, if a researcher asks and receives a police report, parts of the report are redacted. Sometimes, the redacted sections consist of only a few sentences, but more often than not, they are whole paragraphs or sometimes whole pages. One reason given for redacted sections is to protect the identity of the victims, especially when children are involved. Another reason might be to protect otherwise potentially sensitive information. The information redacted also varies by state.

## Police Reports are not Comparable

As was mentioned above, the length of police reports we received ranged from 3 to 182 pages. Needless to say, information contained in these reports was not comparable. The reports differed between different jurisdictions, and within the same jurisdiction, according to individual writer differences. Individual writer differences may be due to one's experience in conducting police reports, time and resources available, and work stress and the number of cases handled, in addition to their varied writing styles. One will not find all the information one is looking for in all of the reports—although most reports mention demographic characteristics. Consequently, research data are not comprehensive and the data set has a lot of missing values, and thereby limits researchers' ability to acquire the same variables, to aggregate data and compare them with each other.

## Obtaining Reports May Be Met With Fees and Charges

As researchers, we expected police reports to be free. We thought we would just contact a police station and ask for a report and we might receive a copy either via email or regular mail. In many cases, the reports were free; in many other cases, the fees were nominal ($2–$6), sometimes the fees were between $10 and $16. There were, however, some police departments that imposed fees of $26 or $30 or $32.50 or $80.02 or $117.00. In most of these instances, the fees involved an hourly rate for services related to producing the report and fees per page. Thus, research involving many police reports can get expensive and may require adequate funding.

## The Quality of the Information Might Not Be as Good as Expected

As was previously stated, for research purposes, we needed a police report to be detailed and to contain accurate information about an incident or crime. This was, however, not always the case. In our experience, what we found was that most police reports focused on the scene of the crime, on the victims, and on witnesses and bystanders. Contrary to what we expected, not much information was provided about the suspect.

Some research studies have also shown problems with the quality of information, for example reporting on lying to the police, even in victimization cases or regarding ethical breaches and unlawful police conduct. Research analyzing over 1,000 police reports in Spain with an automated program VeriPol has shown that people sometimes lie to the police—even if it is a felony or a misdemeanor—thus providing inaccurate information (Quijano-Sánchez et al., 2018). A meta-analysis including seven studies explored the rate of “confirmed false reports of sexual assault to police” (Ferguson and Malouff, 2016, p. 1185). The total rate of false reporting in this analysis exceeded 5%.

A survey study in the Netherlands compared information participants shared about victimization with information from their police reports (Averdijk and Elffers, 2012). In some cases, what the victims expressed first in the survey, was not found in the later police reports; in other cases, information first gathered in the police reports was not found in the survey responses collected later. Overall, findings showed that data of 18% the survey responses on victimization were not consistent with those in the police reports.

The truthfulness of a police report depends on the ethical behavior of the police officer conducting the interviewing, questioning, or interrogation. Minor mistakes might happen and are unintentional. Some mistakes might, however, be deliberate or may be influenced by how much police officers care about following ethical guidelines and protocol. Support for this argument comes from a study of 5,545 sworn law enforcement officers who were arrested during the years 2005 through 2011. These officers were employed by 2,529 non-federal state and local law enforcement agencies in all 50 States of the United States and the District of Columbia (Stinson et al., 2016). They were arrested for five types of crimes: sex-related, alcohol-related, drug-related, violence-related, and profit-motivated crimes. The arrest rate was 0.72 per 1,000 officers or 1.7 officers per 100,000 population nationwide.

## The Reports Might Be Biased

Related to the previous point regarding the quality of information is the potential of skewed information in reports due to bias. Perhaps the following quote from a police report illustrates possible bias: “After canvassing the scene, there were no cooperating witnesses that would provide any possible information.” The report did not describe why witnesses did not cooperate. Were they scared of possible retaliation by the perpetrator? Were they in shock? Did they not trust the police officer? Why? We do not know.

What we are presupposing is the context of bias that influences how police officers conduct their jobs, which may consequently impact writing a police report, which is only one part of what they do. Police officers work under a lot of stress, and are confronted with unforeseen situations on a daily basis (Toch, 2002). What is surprising is that apparently for police officers, departmental politics, and top-down management practices are far more stressful than the dangers in the streets (Toch, 2002).

Research has also shown some biases of police officers, for example in reports related to intimate partner violence (Twis et al., 2018). A study conducted in Hong Kong compared the injury grading in police reports with the trauma records of a regional hospital (Tsui et al., 2009). Findings showed a discordance, with police reports widely overestimating the injury severity. Of course, no one can expect accurate descriptions of medical injuries by police officers. The point we emphasize is that certain information in police reports—in this case the extent of an injury—should be interpreted with caution by researchers.

Another study showed racial bias by police officers not in relation to reports, but to killing unarmed Americans by the police. The probability that an unarmed black American will be shot by the police in the United States is 3.49 times higher than the probability that an unarmed white American will be shot by the police (Ross, 2015).

## Discussion

The goal of this paper was to describe problems related to acquiring police reports and problems related to the quality of police reports as a potential data source for research. We present these problems to alert researchers and to prepare them for using police reports during the research process. Given the problems related to obtaining and analyzing police reports, researchers should explicitly report the response rates of police departments and the number of analyzed reports. That way partial reporting and potential publication bias are made transparent. Moreover, what we have learned too is that police reports may be a valuable source of data, if the data have to do with victim characteristics, descriptions of the scene, witnesses, bystanders, and the details of the shooting incident itself, but not when the data are about the suspect or shooter characteristics.

Having a federal grant supporting such research or having support from the Department of Justice would most likely facilitate the data gathering process. Our experiences when communicating with police departments showed that they were professional and very helpful. That leaves us to surmise that the problem with collecting these data is systematically difficult—that there are institutional procedures in place that keep science or scientific research or other outsiders from meddling into “the way we do things.” For our research purposes, when the data that we gathered from the source (i.e., police reports) were not sufficient, we ended up relying more extensively on other data archives, news outlets, and media coverage.

## Author Contributions

All authors listed have made a substantial, direct and intellectual contribution to the work, and approved it for publication.

## Conflict of Interest

The authors declare that the research was conducted in the absence of any commercial or financial relationships that could be construed as a potential conflict of interest.
